# Feature-weighted maximum representative subsampling

**DOI:** 10.1038/s41598-026-54180-1

**Published:** 2026-06-03

**Authors:** Tony Hauptmann, Stefan Kramer

**Affiliations:** Institute of Computer Science, Johannes Gutenberg University Mainz, Mainz, Germany

**Keywords:** Debiasing, Feature weights, Computational biology and bioinformatics, Engineering, Mathematics and computing

## Abstract

In the social sciences, it is often necessary to debias studies and surveys before valid conclusions can be drawn. Debiasing algorithms enable the computational removal of bias using sample weights. However, an issue arises when only a subset of features is highly biased, while the rest are already representative. Algorithms need to substantially alter the sample distribution to handle a few highly biased features, which can, in turn, introduce bias into otherwise representative variables. To address this issue, we developed a method that uses feature weights to minimize the impact of highly biased features on the computation of sample weights. Our algorithm is based on Maximum Representative Subsampling (MRS), which debiases datasets by iteratively removing elements from a non-representative sample to align it with a representative one. The new algorithm, named feature-weighted MRS (FW-MRS), decreases the emphasis on highly biased features, allowing it to retain more instances for downstream tasks. The feature weights are derived from the feature importance of a domain classifier trained to differentiate between the representative and non-representative datasets. We validated FW-MRS using eight tabular datasets, each of which we artificially biased. Biased features can be important for downstream tasks, and focusing less on them could reduce generalization. For this reason, we assessed the generalization performance of FW-MRS on downstream tasks and found no statistically significant differences. Additionally, FW-MRS was applied to a real-world dataset from the social sciences. The source code is available at https://github.com/kramerlab/FeatureWeightDebiasing.

## Introduction

An ongoing challenge in the social sciences is that a sample may not accurately represent the broader population. When research relies on biased samples, it can lead to invalid conclusions and mistaken inferences about social processes^1^. An example of a biased study is one conducted in a specific city, despite the goal of gathering information about the entire country. If the study’s bias is not addressed, researchers may misrepresent the population and draw inaccurate conclusions^2^.

Bias can occur at any stage of a research project, including study design, data collection, or data analysis^3,4^. The most effective way to minimize bias is to plan the study rigorously. However, even the best planning cannot always prevent it. The bias of a study should not be treated as a dichotomous value but rather as a measure of intensity and type. When collecting additional data is not possible, algorithmic debiasing becomes the preferred option. Selecting an approach that is appropriate to the strength and nature of the bias present can yield more consistent findings and conserve time and resources by reducing the need for repeated studies^5^. Although debiasing algorithms rarely remove bias completely, they can reduce it to a degree where the results remain valid and usable.

In practice, not all features of a dataset are equally biased. While some features may deviate strongly from the target population distribution, others are already representative. Standard debiasing methods treat all features uniformly, which means that correcting a few highly biased features can distort the distribution of otherwise representative ones. Additionally, the strong influence of highly biased features forces these methods to discard many samples, reducing the data available for downstream tasks and thereby weakening the statistical power of subsequent analyses. Feature weights address these issues by reducing the influence of highly biased features during debiasing, preserving the distribution of less-biased features, and retaining more samples, thereby yielding more reliable statistical estimates.

In this work, we develop a method that tackles this challenge by incorporating feature weights into an algorithm that mitigates bias with sample weights. Instead of completely removing highly biased features, which could lead to the loss of valuable information, we propose a “soft” feature selection approach that utilizes feature weights. Our solution is based on *Maximum representative subsampling* (MRS)^6^, which reduces bias by indirectly modeling the distributions of non-representative and representative samples using discriminative machine learning. MRS removes elements from the biased sample that are highly likely to be non-representative, thereby iteratively aligning the distributions and, in turn, generating a representative subsample. It uses uniform weights that avoid assigning high values to samples that would otherwise dominate the analysis.

**Fig. 1:**
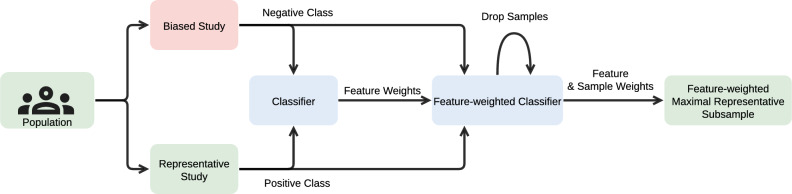
Schema for feature-weighted maximum representative sampling (FW-MRS). Two surveys stem from the same population: one is biased and includes the variable under investigation, while the other is representative but excludes it. FW-MRS mitigates bias by comparing the distributions of the biased and representative datasets using a classifier that leverages information from the representative dataset to remove samples from the biased dataset. The algorithm returns a representative subset and feature weights that align the non-representative study to the distribution of the representative one.

Our method, *Feature-weighted MRS* (FW-MRS), combines MRS with feature weights to reduce the influence of highly biased features, while still addressing the bias in less biased features. This strategy helps mitigate the effects of highly biased features, enabling us to retain more samples. The workflow of FW-MRS is illustrated in Fig. 1.

This paper makes the following contributions: We propose FW-MRS, a novel extension of Maximum Representative Subsampling that incorporates feature weights to reduce the influence of highly biased features on the decision about which samples to drop. Unlike existing methods that treat all features uniformly, FW-MRS uses a softmin transformation of feature importances to perform a soft, data-driven feature selection that retains more samples while still aligning distributions. We present two concrete implementations of the framework: FW-MRS$_{RF}$, based on feature-weighted random forests with SHAP-derived importances, and FW-MRS$_{SVM}$, based on a linear SVM with Linear SHAP, offering a trade-off between modeling flexibility and computational efficiency. We provide a systematic empirical evaluation across eight publicly available tabular datasets, demonstrating that FW-MRS retains more samples than MRS with no statistically significant loss in downstream classification performance. Additionally, we validate FW-MRS on a real-world dataset, showing that the method reduces both the number of dropped samples and the distances between sample distributions relative to a representative reference dataset.

The paper is organized as follows: First, we summarize related work and provide an overview of MRS (“Related Work” section). Next, we introduce and explain the FW-MRS framework, presenting two variations based on different classification algorithms (“Feature-weighted Maximum Representative Subsampling” section) and provide an overview of the used metrics and datasets and the experimental setup (“Evaluation Metrics” to “Experimental Setup” sections). In our experiments, we investigate how the temperature hyperparameter affects the number of dropped samples (“Temperature Analysis” section). We present findings from eight publicly available datasets in which we introduced artificial bias and performed a downstream classification. We validate the impact of the temperature hyperparameter on both the number of dropped samples and the downstream task’s performance across these eight datasets (“Downstream Classification” section). Subsequently, we apply FW-MRS to a real-world biased study and compare its results with those of MRS, focusing on the number of dropped samples and distribution alignment (“Real-world Application” section). Finally, in “Discussion” section we provide a summary and discussion of our results.

## Related work

Bias reduction algorithms exist in a great variety, and most of them rely on different types of additional information, such as class probabilities or computing sampling distributions to make corrections^7,8^. As distributions are rarely known and distribution estimation in high-dimensional spaces is complex, recent methods match distributions between the training and validation sets without performing density estimation.

One approach is to compute a set of sample weights that transform the dataset’s distribution and are used in the downstream task. *Kernel Mean Matching* (KMM)^9^ is a non-parametric method that directly produces sampling weights without distribution estimation by reweighting the samples so that the means of the training and test samples in a reproducing kernel Hilbert space are close. Another algorithm is *propensity score adjustment* (PSA)^10^, which trains a classifier to estimate individuals’ propensities for participation in a non-representative dataset. The most commonly applied method in PSA is logistic regression. However, recent research has shown that different machine learning methods can be advantageous in estimating the propensity score^11^.

A similar task to debiasing is domain adaptation, which addresses domain shifts. One established approach to domain adaptation with complex samples is to map the samples into a potentially lower-dimensional space where instances contain no domain information. Neural Networks are often used for that purpose, especially for images or text, but they are rarely used for tabular data. A well-known example of this is the *Domain-adversarial Neural Network* (DANN)^12^. It is a multi-target model that first embeds the data and splits into two classification branches: one for the domain and the other for the target. The gradient of the domain classification branch is inverted in the feature extractor to encourage training embeddings without domain information while preserving information essential for the target classification.

Not all features are equally representative of the hidden patterns, especially in real-world scenarios. To account for the differing amounts of information each feature provides, feature weights can be applied before model training. The primary advantage of feature weights is their ability to adjust each feature’s influence based on its estimated relevance to the predicted output. Feature weighting methods focus on evaluating a feature’s importance in extracting the underlying pattern^13^.

Feature weights can be applied to samples by multiplication, creating an adjusted dataset that serves as input to a classifier^13^. Alternatively, scaling-invariant algorithms, such as *random forests* (RF), can directly incorporate feature weights without modifying the dataset itself. These weights influence the algorithm’s internal mechanisms. In feature-weighted random forests, the feature weights are integrated into the node-splitting process. In this case, features are selected based on relative weights rather than uniformly, allowing the model to prioritize the most informative features. This adjustment enhances the model’s predictive performance, particularly in high-dimensional data or weak-signal scenarios, such as gene expression data, and when incorporating prior knowledge^14^.

While existing weighting methods primarily focus on sample weighting, they overlook the influence of individual features on the domain shift. By integrating feature weights into the sample reweighting process, FW-MRS improves domain alignment while maintaining predictive performance.

## Methods

### Feature-weighted maximum representative subsampling

The proposed method builds on MRS, which we briefly summarize here (see again Fig. 1). MRS addresses bias in a non-representative dataset (*N*) by leveraging distributional information from a representative dataset (*R*) to remove biased samples and create a representative subsample. The representative dataset must originate from the same population and share as many variables as possible with the biased dataset^6^. Such datasets can be obtained, for example, from public opinion research institutes or governmental statistical agencies that have conducted studies of the same target population using rigorous, unbiased data-collection methods. While these datasets may not be perfectly representative, they are the best approximation in practice. It is important to note that the representative dataset does not contain the required outcome for downstream classification or the dependent or independent variable for a statistical test; if it did, the representative dataset could be used instead. As FW-MRS extends MRS, it also assumes the availability of a representative dataset.

MRS is based on *positive-unlabeled* (PU) learning, a semi-supervised binary classification framework designed to work with only positive and unlabeled data^15^. Pu learning requires two datasets: a positive set *P* and an unlabelled set *U* containing both positive and negative instances. The classifier is trained by treating all samples in *U* as negative, even though some are positive, and is used to predict labels for an unlabeled test set *T*. MRS adapts PU learning by treating the representative dataset *R* as *P*, treating the training set of the biased dataset *N* as *U*, and the remaining portion as the test set *T*. A classifier is trained to distinguish samples from *R* from samples of *N*, without explicitly modeling their distributions. Samples in *T* that the classifier identifies as most likely non-representative are iteratively removed by setting their sample weights to zero. This process continues until the current classifier can no longer distinguish between the datasets, i.e., it predicts no better than random guessing. The final sample weights transform *N* to a subset with approximately the same distribution as *R*^6^.

With this overview in mind, we now introduce the FW-MRS framework. The intention of feature weights is to downweight the influence of highly biased features when dropping samples, since dropping too many samples would be needed to make these feature distributions representative. Instead, feature weights allow the algorithm to focus on mitigating feature bias in lightly biased features. The feature weights also have to be applied in the downstream task; otherwise, it would rely on the still-biased, downweighted features. To compute the feature weights, first, an unweighted domain classifier is trained to distinguish representative from non-representative samples. The domain classifier assigns higher probabilities to overrepresented samples. We assume that features with high predictive importance are exactly those whose distributions differ most from those of the representative dataset, since the classifier uses them to distinguish samples.

For feature weighting, a probability distribution is needed, and highly important features should be assigned low weights, and vice versa. The softmin function provides this transformation from importance to weight:

1 $$\begin{aligned} Softmin(I_i, t) = \frac{e^{\frac{-I_i}{t}}}{\sum _{j} e^{\frac{-I_j}{t}}}, \end{aligned}$$

where $I_i$ denotes the feature importance of feature *i*, and *t* is the temperature hyperparameter. Lower values of *t* produce a more peaked distribution, emphasizing differences in importance, while higher values yield a more uniform weighting.

We now explain the FW-MRS framework in more detail on the basis of the pseudo-code Algorithm 1. The input to FW-MRS consists of the non-representative dataset (*N*), the representative dataset (*R*), the number of cross-validation splits (*k*), and the number of dropped samples per iteration (*d*). Like MRS, FW-MRS outputs sample weights, but additionally returns feature weights. The algorithm begins by initializing all sample weights uniformly (Line 1). A domain classifier is then trained to distinguish between samples from *N* and *R*, and feature importances are computed (Line 2). These importances are converted into feature weights using the softmin function (Line 3).

**Algorithm 1:**
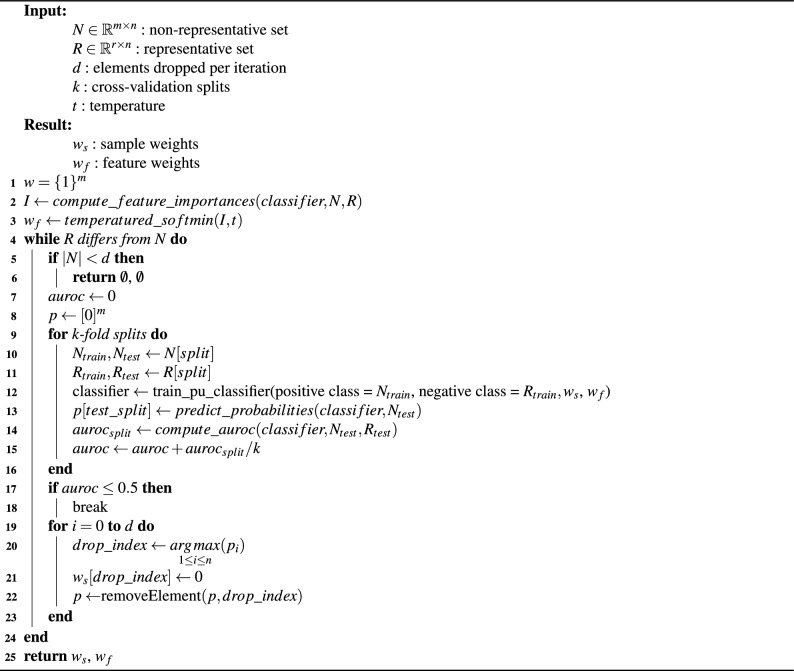
Pseudocode for feature-weighted maximal representative subsampling.

After computing the feature weights, FW-MRS enters the while loop that removes samples until *R* and *N* are no longer distinguishable by the feature-weighted domain classifier. If the threshold is never reached, it indicates that the bias cannot be sufficiently mitigated for use in downstream tasks. This is measured by checking whether the number of remaining samples exceeds the number of samples to be dropped in the next step (Lines 5-6). In this case, the algorithm returns no sample and feature weights.

If it is still possible to drop more samples, the weighted domain classifier is trained to incorporate both the current sample weights and the feature weights. The classifier is trained using *k*-fold cross-validation in conjunction with PU learning. For each fold, the classifier’s outputs on the hold-out sets are stored for later use (Lines 9–13). Additionally, the mean *Area Under the Receiver Operating Characteristic Curve* (AUROC) over folds is computed to evaluate the stopping criterion (Lines 14–15).

If the mean AUROC falls below 0.5, i.e., the classifier is not better than random guessing, FW-MRS terminates, assuming sufficient distribution alignment (Lines 17-18). Another possibility would be to allow the user to set the threshold variable to higher values, but that would introduce another hyperparameter. If the threshold is not reached, the *d* samples most confidently identified as non-representative are removed by setting their weights to zero (Lines 19-22). The process then repeats with updated sample weights. Once the stopping criterion is fulfilled, FW-MRS returns both the sample and feature weights (Line 25). These weights can be used to reweight the non-representative dataset for the downstream task.

For the concrete implementations of the FW-MRS framework, we wanted to employ one classifier exhibiting instance-selection bias and another exhibiting feature-selection bias, yielding FW-MRS$_{RF}$ and FW-MRS$_{SVM}$, respectively. FW-MRS$_{RF}$ employs a *random forest* (RF) as both the domain classifier and the feature importance estimator. It calculates feature importances via SHAP values using TreeShap^16^ with interventional feature perturbation^17^. The weighted domain classifier is implemented as a feature- and sample-weighted random forest. FW-MRS$_{SVM}$ employs a linear *Support Vector Machine* (SVM) with feature importances derived from Linear SHAP^18^. Here, the weighted domain classifier applies feature weights to a linear SVM by scaling the input features of all instances, modifying their influence during training. While the second approach is computationally less demanding, it detects only linear bias, making it suitable for scenarios where bias is expected to be linear or computational efficiency is a priority.

Both classifiers were selected based on their well-understood theoretical properties, a limited number of hyperparameters, and minimal assumptions about the data distribution or intervariable dependencies. Their comparatively low training cost is of practical importance, given that FW-MRS requires numerous classifier runs during both cross-validation and hyperparameter optimization. Because the framework is classifier-agnostic by design, more than the two presented implementations are realizable. More advanced classifiers, such as gradient-boosted trees, which may achieve superior predictive performance, can be used at the possible cost of increased runtime.

Regarding feature importance computation, SHAP values can be slow to compute on large datasets. However, since this computation is performed only once at the beginning of the algorithm, before the iterative sample removal begins, we consider this overhead acceptable for small- to medium-sized datasets. For larger datasets, alternative feature importance measures, such as permutation feature importance or linear SVM coefficients, can be applied within the same framework, each with its own known advantages and disadvantages.

### Datasets

In this section, we introduce the datasets used in the experiments. We validated the approaches on eight tabular datasets, each featuring a binary classification task comprising at least several hundred samples. The datasets span the social and life sciences, where debiasing is most commonly needed. Specifically, we used Folktables Income and Folktables Employment^19^ (hereafter, Income and Employment), Human Analytic, and the Loan dataset from Kaggle. Breast Cancer (Wisconsin), German Credit^20^, Diabetes^21^ and Bank Marketing^22^ from the UCI Repository^23^. The selected datasets are publicly available, contain demographic information, and are presented in a tabular format with a binary classification task. Details about the dataset characteristics can be found in the Supplementary Table 1.

Additionally, a real-world dataset was used to show how FW-MRS can be used by a practitioner. The real-world dataset is part of the *Gutenberg Brain Study* (GBS), a population-based study designed to examine how resilience influences voting behavior. The study was carried out in accordance with relevant guidelines and regulations. The study protocol was approved by the ethics committee of the Rhineland-Palatinate state chamber of physicians (No. 837.085.13, 8770-F), and participants provided written consent. However, a significant limitation of this study is that it was conducted in a university city, which does not accurately reflect the country’s overall demographic diversity. Additionally, the study protocol may be subject to self-selection bias, as individuals with a particular interest in the topic are more likely to participate in the interview.

To mitigate the bias, we used a representative dataset from the Allensbach Institute for Public Opinion Research (Allensbach). To ensure informed consent, participants were informed about the study’s objectives, the procedures for data storage and protection, and their right to withdraw at any time. They were informed that their participation is voluntary. Verbal consent was obtained to ensure anonymity. The interviewers had a standardized questionnaire and could answer further questions, for example, if there were uncertainties. The study was approved by the ethics committee at the Rhineland-Palatinate state chamber of physicians (No 837.209.14, 9448F), and participation in the survey was voluntary. In this dataset, the individuals were selected because they met the criteria for the quota sample, as defined by the German official statistics. Thus, the data can be generalized to the German population with a normal 3% statistical uncertainty in representative surveys.

### Experimental setup

In this section, we describe the baseline methods used for comparison and the procedure for introducing artificial bias into the datasets. We compared FW-MRS to uniform weighting (all samples receive the same weight), the original MRS, and the two most important and widely used sample-weight-based bias-reduction methods: KMM and PSA. For KMM, $\sigma$ was selected heuristically by computing the mean pairwise distance between samples in the combined datasets *N* and *R* using the radial basis function kernel. The second method is PSA with logistic regression, where sample weights are computed as the inverse of the propensity score: $(1 - \pi ) / \pi$^24^. The logistic regression employed $l_2$-regularization, and the regularization parameter *C* was optimized.

Neural network-based methods were not included in the comparison because we focused on tabular data (rather than images or text) and did not want to add a layer of complexity given the expense of hyperparameter optimization. Furthermore, we examine feature and instance weights, which can be inspected and visualized, rather than neural-network-based embeddings or latent representations.

Our evaluation procedure consists of the following steps: First, each dataset is randomly split into five folds for 5-fold cross-validation. The training set is then divided into two equal parts: one is artificially biased to serve as the non-representative dataset *N*, and the other one remains unchanged and acts as the representative dataset *R*. The test set is kept separate and unaltered throughout. To ensure robustness, the entire cross-validation process is repeated 10 times.

Artificial bias is introduced by undersampling the positive class in *N*, retaining only 10% of its original positive samples. This simulates real-world scenarios, such as underdiagnosed medical conditions, misrepresenting the actual prevalence. To keep data sizes manageable, we randomly sampled 6,000 instances from the larger datasets (Diabetes, Employment Income, Bank Marketing, and HR Analytics) before conducting cross-validation. For these datasets, five samples were removed per iteration; for the smaller datasets, one sample was removed per iteration. We empirically select a drop value of 5 samples, which provides an adequate runtime for the larger datasets, given the available time and computing power. A more sophisticated choice would be, e.g., metric-based, such as how much percentage should be dropped per iteration or how many iterations one wants to run the algorithm.

For the downstream task, we use a binary classification on the unbiased test set. The assumption is that downstream targets are available only for *N*, whereas no downstream targets are available for *R* and *T*. This simulates the situation where a biased dataset with classification targets exists, and a representative dataset without them. The task is to train a classifier on a representative dataset without hurting the downstream task too much through debiasing. For the downstream classification, we trained a feature- and sample-weighted random forest with 500 trees, using 5-fold cross-validation on *N* with weights returned by the debiasing methods. The hyperparameters of the downstream classifier and the debiasing were optimized over *N*, using the cross-validation AUROC as the model selection criterion. FW-MRS$_{RF}$ employs a random forest with 200 decision trees and was optimized over the temperature [0.001, 0.0025, 0.005, 0.01, 0.025, 0.05, 0.1, 0.25, 0.5] and the minimum weight fraction per leaf [0.025, 0.01, 0.001, 0.0]. FW-MRS$_{SVM}$ uses a linear SVM with $l_2$ regularization and C was optimized over $[1e-2,..., 1e2]$. In our experiments, the temperature parameter *t* is treated as a hyperparameter and optimized for the downstream task. The final classifier was then used to evaluate performance on the test set.

### Evaluation metrics

In this section, we explain the validation metrics used. To confirm that FW-MRS not only makes the datasets indistinguishable to the classifier used but also increases the similarity of the distributions, the *maximum mean discrepancy* (MMD) is used. MMD measures the distance between two distributions and can be computed as the norm of the difference between the feature means of the distributions in a reproducing kernel Hilbert space^25^.

Given two sets of samples $X= \{x_i\}_{i=1}^n$ and $Y=\{y_i\}_{i=1}^m$, one can compute the empirical estimate of the MMD with sample weights in the following way:

2 $$\begin{aligned} \begin{aligned} \text {MMD}(X, Y, w_X, w_Y, w_f) = \left[ \sum _{i,j=1}^{m} w_{X}^{(i)}w_{X}^{(j)} K(x_i, x_j, w_f) - 2 * \sum _{i,j=1}^{m,n} w_{X}^{(i)}w_{Y}^{(j)} K(x_i, y_j, w_f) + \sum _{i,j=1}^{n} w_{Y}^{(i)}w_{Y}^{(j)} K(y_i, y_j, w_f)\right] ^{\frac{1}{2}} \end{aligned} \end{aligned}$$

where $w_X$ and $w_Y$ are the sample weights, $w_f$ are the feature weights. The kernel *k* is the weighted radial basis function:

3 $$\begin{aligned} k(x, y, w_f) = \exp \left(- \frac{\sum _j w_f^{(j)} (x_j-y_j)^2}{2\sigma ^2}\right). \end{aligned}$$

We determine $\sigma$ heuristically similar to KMM, by setting it to the mean distance between the samples in the aggregated sample, which contains all samples from both datasets^26^.

To assess whether the differences in the downstream task AUROC between MRS and FW-MRS are statistically significant, we performed a corrected repeated *k*-fold cross-validation *t*-test^27^. This test adjusts the variance using a correction factor to account for the inflated Type I error rate introduced by overlapping training sets. Given the multiple comparisons, we adjusted the *p*-values using the Benjamini–Hochberg procedure, which reduces false positives by setting *p*-values to account only for the most reliable results as significant. It reduces false positives by setting *p*-values to consider only the most reliable results significant. We regarded $\alpha < 0.05$ as statistically significant.

## Results

In this section, we present and analyze experimental results comparing FW-MRS with MRS and baseline methods.

### Temperature analysis

**Fig. 2:**
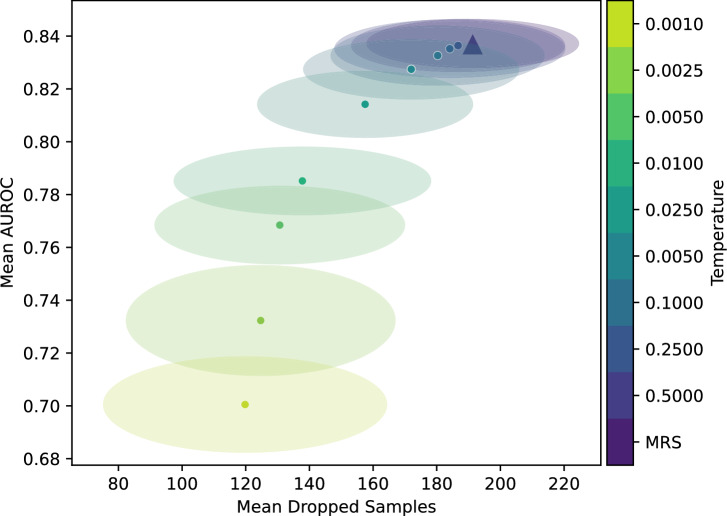
Validation AUROC vs. number of dropped samples: All hyperparameters were fixed except for the temperature. Each point denotes the mean AUROC and dropped samples across runs, with ellipses indicating the standard deviation. Circles correspond to FW-MRS$_{RF}$ and the triangle represents MRS. The temperature changes from high to low, from dark blue to light green.

In this section, we perform two experiments to analyze how the temperature parameter *t* influences the number of dropped samples and downstream classification performance. In the first experiment, we evaluated FW-MRS$_{RF}$ by varying the temperature while keeping all other hyperparameters fixed, and we ran it on Income. For each temperature setting, we compared the number of dropped samples and the downstream AUROC on *R* as a validation metric. Additionally, we performed the same procedure for MRS to work as a baseline without using feature weights at all. The results are shown in Fig. 2, where the mean number of dropped samples is plotted against the mean AUROC. Each point represents the average across the 50 runs, with ellipses indicating standard deviations. Mean results of FW-MRS$_{RF}$ are shown as circles, and the result of MRS as a triangle. The temperature values are shown from high to low, using dark blue to light green.

The diagram illustrates the trade-off between downstream classification performance and the number of dropped samples, as a function of the temperature hyperparameter *t*. Three phases exist: Initially, at a high temperature, as the temperature decreases, both the number of dropped samples and the AUROC decline gradually. This early phase reflects the reduced influence of biased features: although they are weighted less, they are still used sufficiently for downstream classification performance to remain largely unaffected. At high temperatures, the softmin transformation yields nearly uniform feature weights, causing FW-MRS to behave similarly to MRS; both the number of dropped samples and the AUROC remain close to the baseline. This confirms that feature weighting does not harm performance when applied conservatively.

As the temperature is further reduced, feature weighting becomes more extreme, and the AUROC declines more sharply, indicating that informative but biased features are now underutilized. Meanwhile, the number of dropped samples continues to fall at an accelerating rate. This is the most favorable operating region: the number of dropped samples decreases noticeably compared to MRS, while the AUROC remains largely stable.

Eventually, the process reaches a point where most biased features are used infrequently, resulting in only marginal reductions in the number of dropped samples. At this stage, the performance loss outweighs the benefit of retaining more samples. This trade-off is also reflected in the increased variance in AUROC and the number of dropped samples at lower temperatures. At very low temperatures, the weighting becomes extreme, concentrating almost all weight on the single least-biased feature. While the number of dropped samples continues to decline only slowly, the AUROC degrades sharply, and the variance increases, as seen in the larger, more dispersed ellipses. This behavior resembles aggressive feature elimination and suggests that the algorithm is discarding too much discriminative information.

Taken together, Fig. 2 provides a practical guide for temperature selection: intermediate temperatures offer the best balance between sample retention and predictive performance, while very low temperatures should be avoided unless sample preservation is the primary concern at the cost of downstream performance.

**Fig. 3:**
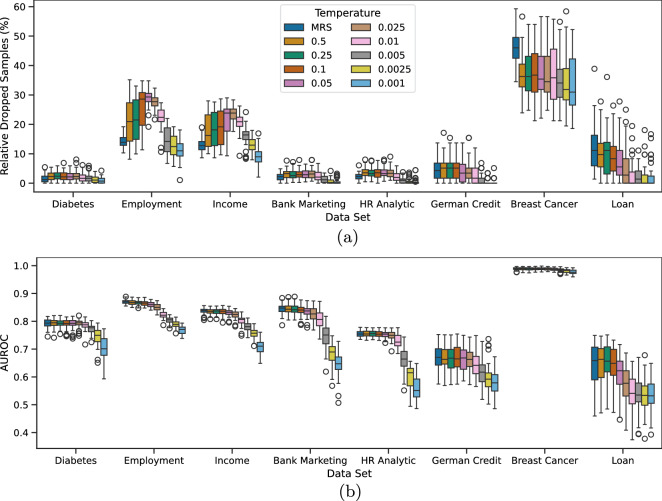
Relative dropped samples and AUROC for 50 iterations with 10 times repeated 5-fold cross-validation for FW-MRS$_{RF}$. Subfigure (**a**) shows the relative dropped samples, and (**b**) the corresponding AUROC. Hyperparameters were optimized with cross-validation over *N* for the downstream classification.

In the second experiment, we analyzed the effect of temperature on the number of dropped samples and downstream classification performance using box plots of the 50 values recorded across iterations for each temperature setting and MRS. Here, the hyperparameters are no longer constant; they are optimized per temperature to achieve the best validation AUROC. Figure 3) shows, on top, the relative dropped samples, and, on the bottom, AUROC.

In many cases, MRS consistently discards the most samples, especially at lower temperatures. In general, incorporating feature weights results in fewer dropped samples, and as the temperature decreases, the number of dropped samples generally declines, though a few exceptions exist. One notable exception with a high rate of dropped samples is Breast Cancer, where all methods tend to drop many samples. This is likely because the dataset has few features, all of which are ordinal, making it challenging to perform debiasing efficiently. The Breast Cancer and Loan datasets illustrate the impact of feature weighting particularly well. Due to its small size and entirely ordinal features, MRS must discard a substantial portion of the data to align the distributions. In contrast, incorporating feature weights significantly reduces the number of dropped samples in these cases.

We also examined the influence of temperature on AUROC. The trend is even more pronounced: lower temperatures lead to a noticeable decline in AUROC, which drops sharply once temperatures fall below a specific threshold. This decline is likely due to the nature of the simulated bias, in which the biased features are also predictive of the downstream classification. At low temperatures, some of these informative features are given minimal weight, causing them to be nearly ignored by the random forest model and effectively removed.

### Downstream classification

After investigating how temperature affects the number of dropped samples and AUROC, we assess FW-MRS’s impact on downstream classification performance and the number of dropped samples by hyperparameterizing all downstream classification parameters. As shown in the previous experiment, a decline in AUROC is expected. However, if the difference is not statistically significant, FW-MRS offers a favorable alternative to existing debiasing methods.

**Fig. 4:**
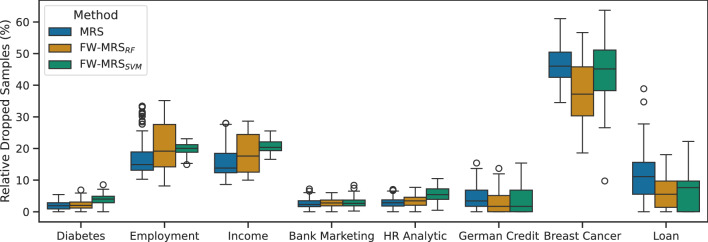
Relative dropped samples over 50 iterations with 10 times repeated 5-fold cross-validation.

First, we visually examine the relative number of dropped samples for MRS and both FW-MRS variants optimized for the downstream classification. Figure 4 displays the box plots along with the corresponding relative number of dropped samples for each method. It is visible that FW-MRS$_{RF}$ retained more samples than MRS in five of eight datasets, while FW-MRS$_{SVM}$ did so in four. Overall, both FW-MRS variants tend to keep more samples, particularly on smaller datasets.

Second, the mean AUROC and standard deviation for all methods for the downstream classification are reported in Table 1. Uniform weighting achieved the best mean ranking, and its strong performance requires some explanation. The artificial bias was introduced by undersampling the positive class, meaning that the most biased features are also the most predictive. As a result, any debiasing method that downweights or removes biased samples simultaneously discards information relevant to the downstream task. Uniform weighting avoids this by leaving the training set unchanged, preserving all predictive information at the cost of leaving the bias uncorrected.

**Table 1:** Downstream classification AUROC over 50 iterations with 10 times repeated 5-fold cross-validation.

Dataset	Uniform	KMM	PSA	MRS	FW-MRS$_{RF}$	FW-MRS$_{SVM}$	Unbiased
Diabetes	$\mathbf {0.791\pm 0.02}$	$0.781\pm 0.02$	$0.787\pm 0.02$	*0.789 ± 0.02*	$0.784\pm 0.02$	$0.782\pm 0.03$	$0.811\pm 0.01$
Employment	$\mathbf {0.871\pm 0.01}$	$0.857\pm 0.01$	$0.867\pm 0.01$	*0.870 ± 0.01*	$0.868\pm 0.01$	$0.864\pm 0.01$	$0.888\pm 0.01$
Income	$\mathbf {0.838\pm 0.01}$	$0.820\pm 0.01$	$0.831\pm 0.01$	*0.837 ± 0.01*	$0.836\pm 0.01$	$0.833\pm 0.01$	$0.866\pm 0.01$
Bank Marketing	$\mathbf {0.847\pm 0.02}$	$0.832\pm 0.03$	$0.839\pm 0.03$	*0.845 ± 0.02*	$0.843\pm 0.02$	$0.840\pm 0.02$	$0.906\pm 0.01$
HR Analytic	$\mathbf {0.753\pm 0.02}$	$0.749\pm 0.02$	$0.750\pm 0.02$	*0.751 ± 0.02*	$0.750\pm 0.02$	*0.751 ± 0.02*	$0.764\pm 0.02$
German Credit	*0.667 ± 0.05*	$0.649\pm 0.05$	$0.659\pm 0.06$	$\mathbf {0.672\pm 0.05}$	$0.642\pm 0.06$	$0.639\pm 0.07$	$0.764\pm 0.03$
Breast Cancer	*0.988 ± 0.01*	$\mathbf {0.989\pm 0.01}$	*0.988 ± 0.01*	$\mathbf {0.989\pm 0.01}$	$0.981\pm 0.01$	$0.977\pm 0.01$	$0.992\pm 0.00$
Loan	$\mathbf {0.658\pm 0.08}$	$0.610\pm 0.10$	$0.628\pm 0.10$	*0.645 ± 0.09*	$0.612\pm 0.08$	$0.586\pm 0.10$	$0.753\pm 0.05$
Rank	**1.44**	5.06	3.88	*1.88*	3.94	4.81	$\emptyset$

The numbers are the means and standard deviations. The best values are written in bold, and the second best is italic. No statistically significant differences between FW-MRS and MRS could be detected. The significance was tested with a corrected *t*-test and the Benjamini-Hochberg procedure. For Unbiased metrics, the downstream classifier was trained on an unbiased dataset.

MRS achieved the second-highest rank, following uniform weighting. PSA and KMM showed the largest decreases in predictive performance, likely due to the assignment of extreme sample weights that significantly altered their influence during training. FW-MRS$_{SVM}$ resulted in a greater reduction in AUROC than FW-MRS$_{RF}$, whose performance remained comparable to that of MRS. These results indicate that although feature weighting can reduce downstream performance, the proposed methods perform competitively. Overall, the mean ranking should be interpreted with caution, as the differences in mean AUROC across methods are minor and the standard deviation intervals largely overlap.

To assess whether the differences between MRS and FW-MRS are statistically significant, we performed a corrected repeated *k*-fold cross-validation *t*-test^27^. Although both FW-MRS variants slightly decreased the mean AUROC, none of the differences were statistically significant. This indicates that incorporating feature weights has a negligible impact on downstream performance in most cases.

Together, the temperature comparison and downstream task validation highlight that, while FW-MRS can retain more samples than MRS, the defined optimization procedure prioritizes performance over sample preservation. If retaining samples is the primary objective, the optimization criterion can be adjusted accordingly, or a compound criterion balancing both objectives can be employed. Reducing the temperature not only reduces the number of dropped samples but also improves distributional alignment, reflected in the reduced MMD between the debiased dataset *N* and the representative test set *T*, as reported in Supplementary Table 4.

### Real-world Application

In this experiment, we apply FW-MRS$_{RF}$ to a real-world case and analyze the influence of the temperature on the number of dropped samples and MMD.

FW-MRS$_{RF}$ was employed to minimize bias in GBS, dropping 1 sample per iteration. The debiasing was repeated 50 times, and the mean feature importance of the unweighted feature importances and the mean of the derived feature weights were calculated. Additionally, the mean number of dropped samples per temperature and the MMD between the weighted GBS and Allensbach were computed. The hyperparameters of FW-MRS$_{RF}$, except the temperature, were optimized to minimize the MMD between GBS and Allensbach. We also tracked the mean AUROC per iteration across runs and plotted it in Supplemental Fig. 1.

**Fig. 5:**
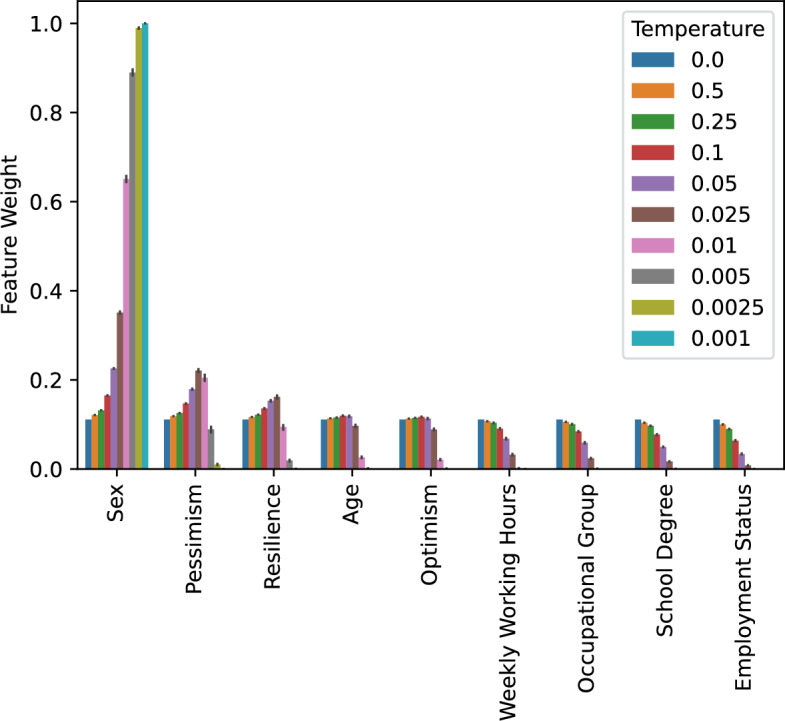
Feature weights used in FW-MRS$_{RF}$ debiasing of GBS with information of Allensbach.

The feature weights provide a way to measure the degree of bias in each feature, and the mean feature weights with standard deviation are visualized in Fig. 5 to provide an overview (see the Supplemental Fig. 2 for a visualization of the feature importances). The features are ordered from left to right in ascending order of feature importance, i.e., from least biased to most. As expected, employment status, educational attainment, and occupational group are the most biased features, given the city’s higher educational level. For FW-MRS$_{RF}$, it starts with a nearly uniform distribution, similar to MRS. However, as the temperature decreases, the weights shift toward features with the lowest importance until, at some point, almost all the weight is assigned to the least important feature.

Table 2 contains the mean MMD and number of dropped samples for FW-MRS$_{RF}$ with different temperatures and MRS. As the temperature decreases, more samples are retained, and the MMD between GBS and Allensbach decreases. However, choosing the lowest temperature is not advisable, as it assigns feature weights primarily to a single feature (sex), which is not informative on its own.

**Table 2:** Mean number of dropped samples and MMD of 50 runs debiasing of GBS with information of Allensbach with FW-MRS$_{RF}$.

Temperature	Number of Dropped Samples	MMD
MRS	$501.1 \pm 4.9$	$0.153 \pm 0.004$
0.5	$501.0 \pm 3.6$	$0.151 \pm 0.004$
0.25	$497.5 \pm 3.6$	$0.145 \pm 0.003$
0.1	$493.2 \pm 4.2$	$0.132 \pm 0.005$
0.05	$478.7 \pm 5.1$	$0.114 \pm 0.004$
0.025	$444.5 \pm 4.9$	$0.086 \pm 0.002$
0.01	$305.8 \pm 12.7$	$0.059 \pm 0.001$
0.005	$235.8 \pm 15.3$	$0.039 \pm 0.002$
0.0025	$152.2 \pm 9.7$	$0.034 \pm 0.002$
0.001	$143.0 \pm 7.6$	$0.032 \pm 0.001$

The choice of the temperature *t* is substantial for the algorithm because it strongly influences the computed feature weights and, in turn, the sample weights. There exists a trade-off between the number of dropped samples and the MMD, while accounting for the weights assigned to different features. We recommend that practitioners at least perform hyperparameter optimization using the hyperparameter grid we used in our experiments. If the minimum required number of samples is known in advance, one can start with a high temperature and gradually reduce it until the remaining number of samples reaches that minimum.

## Discussion

In this paper, we introduced the FW-MRS framework and presented two specific implementations: one based on random forests (FW-MRS$_{RF}$) and the other on linear SVMs (FW-MRS$_{SVM}$). FW-MRS extends the original MRS approach by incorporating feature weights that control the influence of biased features during both downsampling and downstream classification.

Before discussing our findings, we briefly summarize the key results:

- There is no evidence that the downstream performance of FW-MRS is statistically significantly different from that of MRS, the currently best method based on downstream AUROC (Table 1).
- FW-MRS retains more instances and thus reduces, in some cases, the variance component of the error compared to MRS (see the lower part of Supplementary Table 2).
- Considering the distribution alignment, FW-MRS improves on MRS (see Supplementary Table 4). While KMM and PSA achieve even stronger alignment, this comes at the cost of a greater decline in downstream classification performance (Table 1), suggesting a less favorable trade-off.
- Temperature selection is critical, and can be supported with visualizations similar to Fig. 2 or determined via hyperparameter optimization.

The iterative subsampling procedure requires repeated classifier training across multiple cross-validation folds and increasing computational cost as the dataset size grows. It is therefore best suited to small and medium-sized datasets, and practitioners working with large-scale data are advised to consider more computationally efficient variants such as FW-MRS$_{SVM}$. Developing strategies for larger datasets is a promising direction for future work.

By downweighting highly biased features, FW-MRS reduces the number of samples that must be discarded to achieve distributional alignment. Among the two implementations, FW-MRS$_{RF}$ had a smaller impact on downstream task performance than FW-MRS$_{SVM}$, making it the preferable choice when predictive performance is the primary concern. High temperatures were mainly used when optimizing hyperparameters for downstream classification to minimize their effect on downstream task quality. However, if a fixed number of samples should be retained, for instance, to maintain adequate statistical power, the optimization objective can be adjusted to prioritize the number of dropped samples or balance both.

FW-MRS is relevant in settings where aligning disparate data distributions is essential to train representative, generalizable models. For instance, in healthcare, datasets are collected mostly independently by institutions and may vary substantially. FW-MRS provides an approach for aligning data from diverse sources, thereby creating more reliable predictive models. FW-MRS can be used to align the datasets, enabling their combination in the downstream task and potentially improving predictive performance. When targets are available for both datasets, this information could be further integrated into the method to enhance alignment.

The FW-MRS framework is designed to be flexible. It can be customized to specific needs by replacing the classifier, redefining feature-importance measures, or adapting the hyperparameter optimization strategy. The method’s adaptability enables more representative and reliable interpretation from datasets affected by strongly biased features.

## Supplementary Information

Supplementary Information.

## Data Availability

GBS and Allensbach are private datasets. Human Resource Analytics (https://www.kaggle.com/datasets/arashnic/hr-analytics-job-change-of-data-scientists) and Loan (https://www.kaggle.com/datasets/burak3ergun/loan-data-set) are freely available on Kaggle. Folktables can be accessed through its Python package (https://github.com/socialfoundations/folktables). Breast Cancer (Wisconsin) (https://archive.ics.uci.edu/dataset/15/breast+cancer+wisconsin+original), German Credit (https://archive.ics.uci.edu/dataset/144/statlog+german+credit+data), Diabetes (https://archive.ics.uci.edu/dataset/34/diabetes), and Bank Marketing (https://archive.ics.uci.edu/dataset/222/bank+marketing) are available at the UCI Repository.

## Code availability

The source code for preprocessing GBS and Allensbach (https://github.com/laksannathan/maximal-representative-sampling) and the methods and experiments (https://github.com/kramerlab/FeatureWeightDebiasing) are available.

## References

[1] Winship, C. & Mare, R. D. Models for sample selection bias. *Annu. Rev. Sociol.* **18**, 327–350. 10.1146/annurev.so.18.080192.001551 (1992).

[2] West, B. T., Sakshaug, J. W. & Aurelien, G. A. S. How big of a problem is analytic error in secondary analyses of survey data?. *PLOS ONE* **11**, e0158120. 10.1371/journal.pone.0158120 (2016). 27355817 10.1371/journal.pone.0158120 PMC4927119

[3] Smith, L. H. Selection mechanisms and their consequences: Understanding and addressing selection bias. *Curr. Epidemiol. Rep.* **7**, 179–189. 10.1007/s40471-020-00241-6 (2020).

[4] Infante-Rivard, C. & Cusson, A. Reflection on modern methods: Selection bias–a review of recent developments. *Int. J. Epidemiol.* **47**, 1714–1722. 10.1093/ije/dyy138 (2018). 29982600 10.1093/ije/dyy138

[5] Keeble, C., Law, G. R., Barber, S. & Baxter, P. D. Choosing a method to reduce selection bias: A tool for researchers. *Open J. Epidemiol.* **5**, 155–162. 10.4236/ojepi.2015.53020 (2015).

[6] Hauptmann, T., Fellenz, S., Nathan, L., Tüscher, O. & Kramer, S. Discriminative machine learning for maximal representative subsampling. *Sci. Rep.* **13**, 20925. 10.1038/s41598-023-48177-3 (2023). 38017053 10.1038/s41598-023-48177-3 PMC10684887

[7] Huang, J., Zhou, J. & Zheng, L. Support vector machine classification algorithm based on relief-F feature weighting. In *2020 International conference on computer engineering and application (ICCEA)*, 547–553, 10.1109/ICCEA50009.2020.00121 (IEEE, 2020).

[8] Dudík, M., Phillips, S. & Schapire, R. E. Correcting sample selection bias in maximum entropy density estimation. In *Advances in Neural Information Processing Systems*, vol. 18 (MIT Press, 2005).

[9] Huang, J., Gretton, A., Borgwardt, K., Schölkopf, B. & Smola, A. correcting sample selection bias by unlabeled data. In *Advances in Neural Information Processing Systems*, vol. 19 (MIT Press, 2006).

[10] Rosenbaum, P. R. The central role of the propensity score in observational studies for causal effects. In *Matched sampling for causal effects* (ed. Rubin, D. B.) 170–184 (Cambridge University Press, 2006). 10.1017/CBO9780511810725.016.

[11] Rueda, M. & d. M., Pasadas-del-Amo, S., Rodríguez, B. C., Castro-Martín, L. & Ferri-García, R,. Enhancing estimation methods for integrating probability and nonprobability survey samples with machine-learning techniques. An application to a Survey on the impact of the COVID-19 pandemic in Spain. *Biom. J.* **65**, 2200035. 10.1002/bimj.202200035 (2023). 10.1002/bimj.202200035 PMC9538074 36136044

[12] Ganin, Y. *et al.* Domain-adversarial training of neural networks, 10.48550/arXiv.1505.07818 (2016). arXiv:1505.07818.

[13] Niño-Adan, I., Manjarres, D., Landa-Torres, I. & Portillo, E. Feature weighting methods: A review. *Expert. Syst. Appl.* **184**, 115424. 10.1016/j.eswa.2021.115424 (2021).

[14] Amaratunga, D., Cabrera, J. & Lee, Y.-S. Enriched random forests. *Bioinformatics (Oxford, England)* **24**, 2010–2014. 10.1093/bioinformatics/btn356 (2008). 18650208 10.1093/bioinformatics/btn356

[15] Bekker, J. & Davis, J. Learning from positive and unlabeled data: A survey. *Mach. Learn.* **109**, 719–760. 10.1007/s10994-020-05877-5 (2020).

[16] Lundberg, S. M. et al. From local explanations to global understanding with explainable AI for trees. *Nat. Mach. Intell.* **2**, 56–67. 10.1038/s42256-019-0138-9 (2020). 32607472 10.1038/s42256-019-0138-9 PMC7326367

[17] Janzing, D., Minorics, L. & Bloebaum, P. Feature relevance quantification in explainable AI: A causal problem. In *Proceedings of the twenty third international conference on artificial intelligence and statistics*, 2907–2916 (PMLR, 2020).

[18] Lundberg, S. & Lee, S.-I. A unified approach to interpreting model predictions, 10.48550/arXiv.1705.07874 (2017). arXiv:1705.07874.

[19] Ding, F., Hardt, M., Miller, J. & Schmidt, L. Retiring adult: New datasets for fair machine learning. *Adv. Neural Inf. Process. Syst.* **34**, 6478–6490 (2021).

[20] Hofmann, H. Statlog (German Credit Data, 1994). https://doi.org/10.24432/C5NC77

[21] Kahn, M. *Diabetes*10.24432/C5T59G.

[22] Moro, S. & Rita, P. Bank marketing (2014). https://doi.org/10.24432/C5K306.

[23] Wolberg, W., Mangasarian, O., Street, N. & Street, W. Breast cancer wisconsin (diagnostic). UCI Machine Learning Repository, 10.24432/C5DW2B (1995).

[24] Schonlau, M. & Couper, M. P. Options for conducting web surveys. *Stat. Sci.* **32**, 279–292. 10.1214/16-STS597 (2017).

[25] Berlinet, A. & Thomas-Agnan, C. *Reproducing Kernel Hilbert spaces in probability and statistics* (Springer, 2011).

[26] Gretton, A., Borgwardt, K. M., Rasch, M. J., Schölkopf, B. & Smola, A. A Kernel two-sample test. *J. Mach. Learn. Res.* **13**, 723–773 (2012).

[27] Bouckaert, R. R. & Frank, E. Evaluating the replicability of significance tests for comparing learning algorithms. In Kanade, T. *et al.* (eds.) *Advances in knowledge discovery and data mining*, vol. 3056, 3–12, 10.1007/978-3-540-24775-3_3 (Springer, 2004).

