# Supplementary material for: Feature-weighted maximum representative subsampling
Source: Sci Rep. 2026 Jun 3;16:17166. doi: 10.1038/s41598-026-54180-1 (PMC13234176; doi:10.1038/s41598-026-54180-1)
Supplement: Supplementary file 1 — Supplementary Information. [file 41598_2026_54180_MOESM1_ESM.pdf]

Feature-Weighted Maximum Representative Subsampling

Tony Hauptmann[1,*] **and Stefan Kramer**[1]

[1]Institute of Computer Science, Johannes Gutenberg University Mainz, Mainz, Germany
[*]Corresponding author: thauptmann@uni-mainz.de

ABSTRACT

In the social sciences, it is often necessary to debias studies and surveys before valid conclusions can be drawn. Debiasing algorithms enable the computational removal of bias using sample weights. However, an issue arises when only a subset of features is highly biased, while the rest are already representative. Algorithms need to substantially alter the sample distribution to handle a few highly biased features, which can, in turn, introduce bias into otherwise representative variables. To address this issue, we developed a method that uses feature weights to minimize the impact of highly biased features on the computation of sample weights. Our algorithm is based on Maximum Representative Subsampling (MRS), which debiases datasets by iteratively removing elements from a non-representative sample to align it with a representative one. The new algorithm, named feature-weighted MRS (FW-MRS), decreases the emphasis on highly biased features, allowing it to retain more instances for downstream tasks. The feature weights are derived from the feature importance of a domain classifier trained to differentiate between the representative and non-representative datasets. We validated FW-MRS using eight tabular datasets, each of which we artificially biased. Biased features can be important for downstream tasks, and focusing less on them could reduce generalization. For this reason, we assessed the generalization performance of FW-MRS on downstream tasks and found no statistically significant differences. Additionally, FW-MRS was applied to a real-world dataset from the social sciences. The source code is available at https://github.com/kramerlab/FeatureWeightDebiasing.

A Dataset Characteristics

Supplementary Table 1. Dataset characteristics.

Name	#Samples	#Positive	#Negative	#Features
Diabetes	253680	35346	218334	21
Folktables Employment	378817	172803	206014	99
Folktables Income	195665	85189	110476	69
Bank Marketing	45211	5289	39922	16
Human Research Analytic	8955	1483	7472	33
Allensbach	1082	∅	∅	54
German Credit	1000	300	700	20
Breast Cancer	683	444	239	10
GBS	579	550	29	52
Loan	480	332	148	13

B Bias-Variance Decomposition

Next, we analyze the bias-variance decomposition of the 0-1 loss. To perform the decomposition, the test procedure was modified: First, each dataset was randomly split into two equal parts: one half referred to as the "world", with a size of $2m$, was used to draw training samples from, while the other half was used as a fixed test set. From the world, we sampled m samples without replacement to form a training set. We introduced a bias within each sample by selecting a non-representative subset N, with the remaining instances forming the representative dataset R. This sampling approach allows $\binom{2n}{n}$ distinct datasets, providing sufficient variety[1]. The sampling was repeated 50 times, but the test set was kept constant to facilitate the bias-variance decomposition (Table 2).

The bias and variance of the 0-1 loss were computed in the following: Given the main prediction $E[\hat{y}]$ as the mode of the predicted classes and $L(y,\hat{y})$ defines the 0-1 loss, the bias is computed as $L(y,E[\hat{y}])$ and the variance as $E[L(\hat{y},E[\hat{y}])]$ with the expectation taken over training sets[2].

Supplementary Table 2. Downstream task bias and variance for 0-1 loss over 50 iterations using repeated resampling. The numbers are the means, the best values are written in bold, and the second best is underlined. No value is underlined in rows where all methods perform equally.

Metric	Dataset	Uniform	KMM	PSA	MRS	FW-MRS$_{RF}$	FW-MRS$_{SVM}$	Unbiased
Bias	Diabetes	**0.135**	**0.135**	**0.135**	**0.135**	**0.135**	**0.135**	0.208
	Employment	**0.212**	0.262	0.213	0.216	0.229	0.224	0.205
	Income	0.292	**0.252**	0.288	0.278	0.291	0.286	0.208
	Bank Marketing	**0.116**	**0.116**	**0.116**	**0.116**	**0.116**	**0.116**	0.097
	HR Analytic	0.165	0.165	0.165	**0.155**	0.165	0.165	0.165
	German Credit	**0.299**	**0.299**	**0.299**	**0.299**	**0.299**	**0.299**	0.257
	Breast Cancer	0.048	**0.031**	0.035	0.035	0.044	0.039	0.018
	Loan	**0.694**	**0.694**	**0.694**	**0.694**	**0.694**	**0.694**	0.206
Rank		3.88	3.25	3.31	**2.81**	4.13	3.63	∅
Variance	Diabetes	0.076	**0.053**	0.094	0.070	0.080	0.084	0.105
	Employment	0.157	0.200	**0.143**	0.160	0.148	0.161	0.029
	Income	0.134	0.256	0.217	**0.133**	0.143	0.141	0.052
	Bank Marketing	0.014	0.034	0.027	0.014	0.013	**0.012**	0.025
	HR Analytic	0.072	0.157	0.109	0.089	0.075	**0.071**	0.046
	German Credit	**0.014**	0.020	0.025	0.020	0.017	0.037	0.147
	Breast Cancer	0.104	0.077	0.040	**0.017**	0.055	0.052	0.007
	Loan	0.066	0.091	**0.064**	0.066	0.092	0.082	0.034
Rank		2.88	4.81	3.75	**2.69**	3.38	3.50	∅

MRS achieves the greatest reduction in both bias and variance, although all methods exhibit the same bias across four datasets. PSA and KMM tend to increase variance due to variability introduced by potentially large differences in sample weights. In contrast, (FW)-MRS assigns uniform weights to all remaining samples, mitigating this effect. However, both FW-MRS variants exhibit increased bias because the downstream classifier places less weight on biased but informative features. FW-MRS decreases the variance in some cases and increases it in others.

C Matthews Correlation Coefficient for Downstream Classification

Table 3 reports the mean *Matthews correlation coefficient* (MCC) and standard deviation with a threshold of 0.5 for the downstream classification across all 50 iterations. The MCC results reveal more pronounced differences between biased and unbiased training conditions than those observed for the AUROC, highlighting the sensitivity of the MCC to the class imbalance introduced by artificial bias. Consistent with the AUROC results, KMM and PSA reduce the MCC compared with uniform weighting. FW-MRS$_{SVM}$ similarly reduces the MCC, while FW-MRS$_{RF}$ and MRS show smaller but comparable reductions. Across all methods, the standard deviation is notably high, reflecting the substantial variability in performance across datasets and runs.

Supplementary Table 3. Downstream classification MCC over 50 iterations with 10 times repeated 5-fold cross-validation. The numbers are the means and standard deviations. The best values are written in bold, and the second best is underlined. For Unbiased metrics, the downstream classifier was trained on an unbiased dataset. No statistically significant differences between FW-MRS and MRS could be detected. The significance was tested with a corrected *t*-test and the Benjamini-Hochberg procedure.

Dataset	Uniform	KMM	PSA	MRS	FW-MRS$_{RF}$	FW-MRS$_{SVM}$	Unbiased
Diabetes	**0.137 ± 0.16**	0.109 ± 0.14	0.124 ± 0.15	<u>0.129 ± 0.15</u>	0.116 ± 0.15	0.121 ± 0.14	0.314 ± 0.09
Employment	0.506 ± 0.14	0.457 ± 0.11	**0.535 ± 0.08**	0.506 ± 0.11	<u>0.517 ± 0.09</u>	0.500 ± 0.12	0.613 ± 0.02
Income	0.397 ± 0.15	0.383 ± 0.11	0.346 ± 0.20	<u>0.402 ± 0.14</u>	**0.407 ± 0.13**	**0.407 ± 0.13**	0.563 ± 0.02
Bank Marketing	0.082 ± 0.13	0.083 ± 0.13	0.063 ± 0.12	0.081 ± 0.13	<u>0.084 ± 0.14</u>	**0.090 ± 0.13**	0.414 ± 0.06
HR Analytic	**0.183 ± 0.21**	0.118 ± 0.17	0.111 ± 0.15	<u>0.171 ± 0.21</u>	0.162 ± 0.21	0.142 ± 0.19	0.353 ± 0.15
German Credit	<u>0.030 ± 0.07</u>	0.008 ± 0.03	0.024 ± 0.06	**0.034 ± 0.08**	**0.034 ± 0.06**	0.024 ± 0.06	0.284 ± 0.10
Breast Cancer	0.781 ± 0.29	0.844 ± 0.22	<u>0.860 ± 0.18</u>	**0.910 ± 0.040**	0.828 ± 0.07	0.814 ± 0.14	0.927 ± 0.03
Loan	0.057 ± 0.09	0.068 ± 0.09	**0.081 ± 0.09**	<u>0.072 ± 0.10</u>	0.053 ± 0.10	0.042 ± 0.08	0.442 ± 0.09
Rank	3.31	4.63	3.69	**2.50**	<u>3.00</u>	3.88	∅

D Distribution Alignment

In this experiment, the feature- and sample-weighted MMD between the debiased non-representative dataset N and the representative test set T is computed and compared across different methods. As expected, uniform weighting yielded the worst results (Table 4). The remaining methods demonstrate varying degrees of improved distribution alignment. MRS achieved moderate alignment, whereas both FW-MRS variants, especially FW-MRS$_{SVM}$, further reduce the MMD, indicating that feature weights improve the distribution alignment. PSA and KMM achieved the lowest MMD values, with KMM achieving the lowest due to its explicit optimization for MMD. However, this came at the cost of a greater decline in downstream task performance in KMM than in MRS and FW-MRS.

Supplementary Table 4. MMD over 50 iterations with 10 times repeated 5-fold cross-validation. The numbers are the means and standard deviations. The best values are written in bold, and the second best is underlined.

Dataset	Uniform	KMM	PSA	MRS	FW-MRS$_{RF}$	FW-MRS$_{SVM}$	Unbiased
Diabetes	0.0358 ± 0.006	**0.0217 ± 0.003**	<u>0.0220 ± 0.003</u>	0.0325 ± 0.005	0.0319 ± 0.005	0.0308 ± 0.006	0.0220 ± 0.003
Employment	0.0681 ± 0.005	**0.0219 ± 0.002**	<u>0.0240 ± 0.002</u>	0.0472 ± 0.013	0.0465 ± 0.014	0.0329 ± 0.005	0.0224 ± 0.002
Income	0.0640 ± 0.005	**0.0233 ± 0.003**	<u>0.0262 ± 0.002</u>	0.0501 ± 0.011	0.0514 ± 0.011	0.0342 ± 0.003	0.0230 ± 0.003
Bank Marketing	0.0282 ± 0.003	**0.0226 ± 0.002**	<u>0.0230 ± 0.002</u>	0.0270 ± 0.002	0.0269 ± 0.003	0.0273 ± 0.003	0.0226 ± 0.002
HR Analytic	0.0276 ± 0.003	**0.0184 ± 0.002**	<u>0.0187 ± 0.002</u>	0.0258 ± 0.003	0.0246 ± 0.003	0.0251 ± 0.003	0.0193 ± 0.002
German Credit	0.0640 ± 0.007	**0.0549 ± 0.006**	<u>0.0561 ± 0.007</u>	0.0615 ± 0.006	0.0601 ± 0.007	0.0631 ± 0.010	0.0565 ± 0.006
Breast Cancer	0.3949 ± 0.017	**0.0429 ± 0.009**	<u>0.1423 ± 0.024</u>	0.2805 ± 0.032	0.2701 ± 0.043	0.2256 ± 0.073	0.0474 ± 0.006
Loan	0.1413 ± 0.015	**0.0803 ± 0.014**	<u>0.1004 ± 0.015</u>	0.1275 ± 0.016	0.1122 ± 0.028	0.1105 ± 0.020	0.0763 ± 0.014
Rank	6.0	**1.0**	<u>2.0</u>	4.63	3.75	3.63	∅

E AUROC per Iteration for GBS

This section examines the changes in AUROC over iterations and compares them across different temperatures. The mean and standard deviation for 50 repeated runs are shown. The diagram (Figure 1) shows that fewer samples have to be dropped using FW-MRS$_{RF}$ compared to MRS. Using lower weights further reduces the number of dropped samples, starting slowly at the beginning and gradually increasing in strength later. The diagram illustrates the potential number of samples that can be retained by incorporating feature weights into the debiasing procedure.

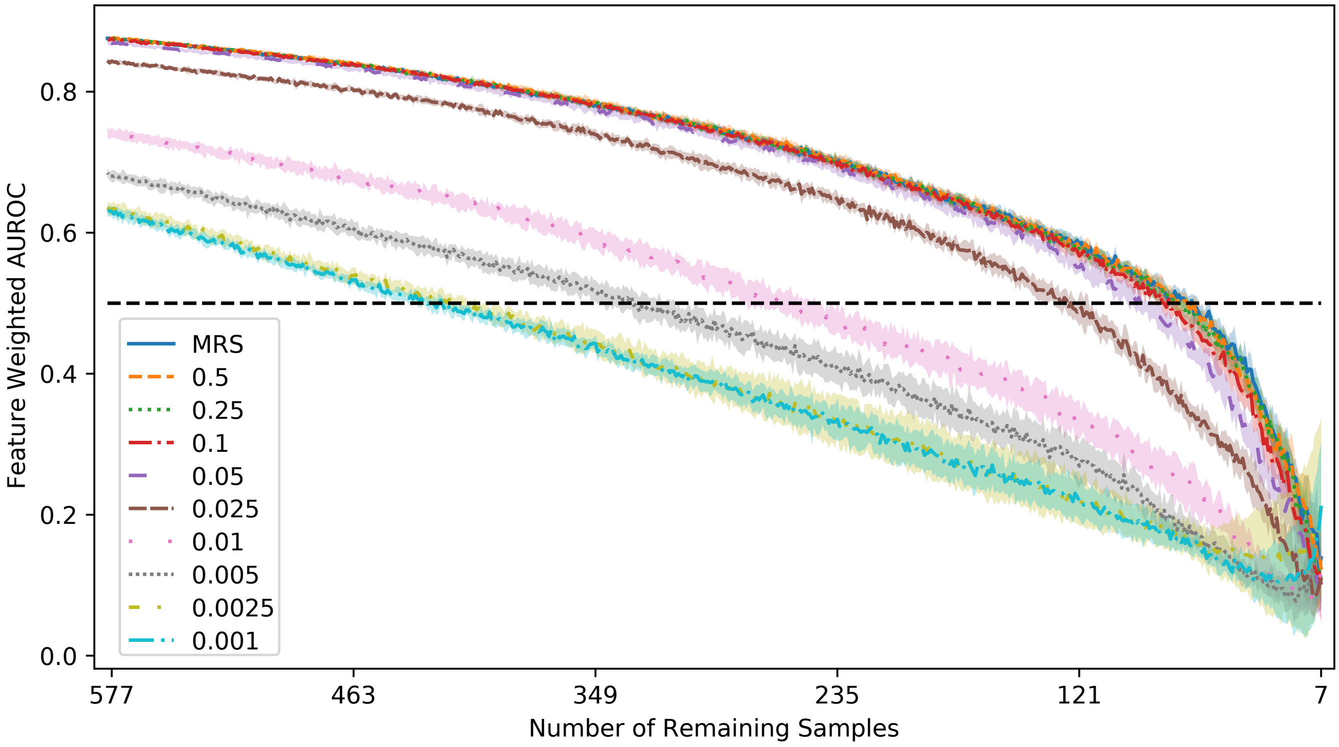

Supplementary Figure 1. Comparison of mean AUROC of MRS vs. FW-MRS$_{RF}$ with different temperatures on 50 debiasing runs of GBS with auxiliary information of Allensbach.

F Feature Importance GBS

Figure 2 illustrates the feature importance of the unweighted domain classifier for GBS and Allensbach. It reveals that the most important and, hence, most biased features are associated with subjects' education and employment. This finding confirms the bias towards an overrepresentation of highly educated individuals.

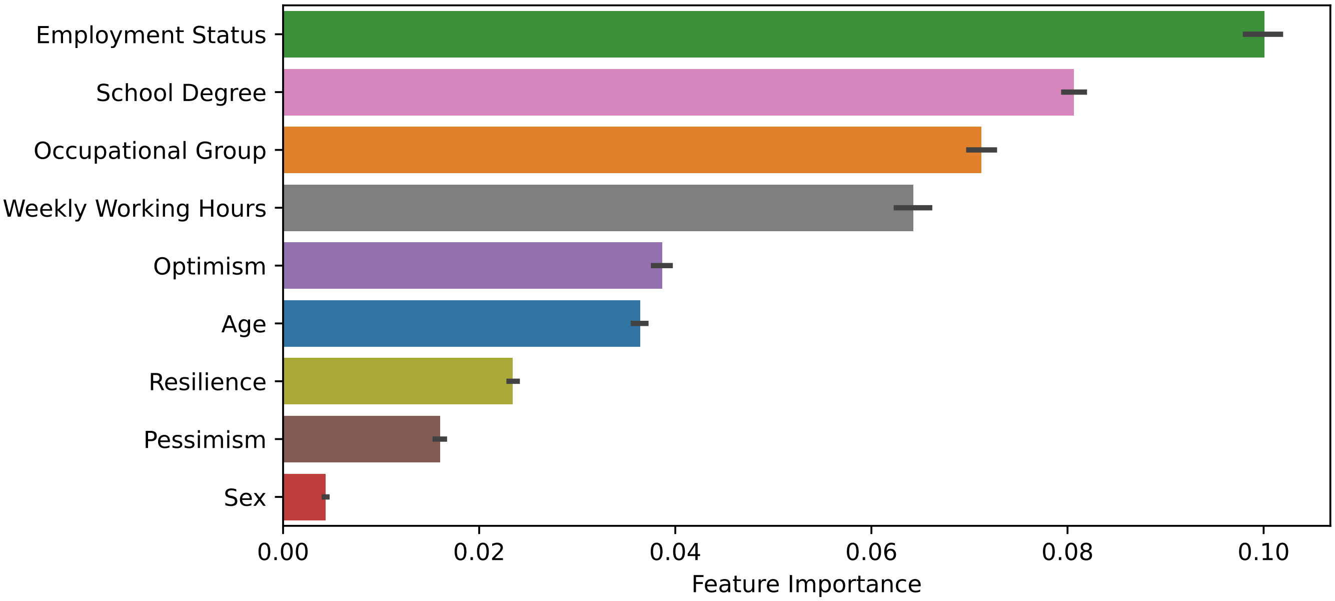

Supplementary Figure 2. Feature importance used in FW-MRS$_{RF}$ debiasing of GBS with auxiliary information of Allensbach.

References

1. Kohavi, R. & Wolpert, D. Bias plus variance decomposition for zero-one loss functions. In *Proceedings of the Thirteenth International Conference on International Conference on Machine Learning*, ICML'96, 275–283 (Morgan Kaufmann Publishers Inc., San Francisco, CA, USA, 1996).

2. Domingos, P. A Unified Bias-Variance Decomposition for Zero-One and Squared Loss. In *Proceedings of the Seventeenth National Conference on Artificial Intelligence and Twelfth Conference on Innovative Applications of Artificial Intelligence*, 564–569 (AAAI Press, 2000).
